# Defining the ethical considerations surrounding kidney transplantation for frail and cognitively impaired patients: a Delphi study of geriatric transplant experts

**DOI:** 10.1186/s12877-022-03209-x

**Published:** 2022-07-08

**Authors:** Prakriti Shrestha, Sarah E. Van Pilsum Rasmussen, Elizabeth A. King, Elisa J. Gordon, Ruth R. Faden, Dorry L. Segev, Casey Jo Humbyrd, Mara McAdams-DeMarco

**Affiliations:** 1 Department of Surgery, Johns Hopkins University School of Medicine, Baltimore, MD USA; 2 Center for Health Services and Outcomes Research, Northwestern University Feinberg School of Medicine, Chicago, IL USA; 3 Department of Surgery, Northwestern University Feinberg School of Medicine, Chicago, IL USA; 4 Johns Hopkins Berman Institute of Bioethics, Baltimore, MD USA; 5 Health Policy and Management, Johns Hopkins Bloomberg School of Public Health, Baltimore, MD USA; 6 Department of Orthopaedic Surgery, University of Pennsylvania, Philadelphia, PA USA; 7 Department of Epidemiology, Johns Hopkins Bloomberg School of Public Health, Baltimore, MD USA; 8 Department of Surgery, New York University, New York, USA

**Keywords:** Bioethics, Frailty, Cognition, Transplantation

## Abstract

**Background:**

Among adult kidney transplant (KT) candidates, 21% are frail and 55% have cognitive impairment, increasing the risk of pre- and post-KT mortality. Centers often assess frailty status and cognitive function during transplant evaluation to help identify appropriate candidate. Yet, there are no ethical guidelines regarding the use of frailty and cognitive function during this evaluation. We seek to develop a clinical consensus on balancing utility and justice in access to KT for frail and cognitively impaired patients.

**Methods:**

Twenty-seven experts caring for ESRD patients completed a two-round Delphi panel designed to facilitate consensus (> 80% agreement).

**Results:**

Experts believed that denying patients transplantation based solely on expected patient survival was inequitable to frail or cognitively impaired candidates; 100% agreed that frailty and cognitive impairment are important factors to consider during KT evaluation. There was consensus that health related quality of life and social support are important to consider before waitlisting frail or cognitively impaired patients. Experts identified important factors to consider before waitlisting frail (likely to benefit from KT, frailty reversibility, age, and medical contraindications) and cognitively impaired (degree of impairment and medication adherence) patients.

**Conclusions:**

Clinical experts believed it was ethically unacceptable to allocate organs solely based on patients’ expected survival; frailty and cognitive impairment should be measured at evaluation when weighed against other clinical factors. Ethical guidelines regarding the use of frailty and cognitive function during KT evaluation ought to be developed.

**Supplementary Information:**

The online version contains supplementary material available at 10.1186/s12877-022-03209-x.

## Background

Only a quarter of patients who were on the kidney transplant (KT) waiting list received a transplant in 2020 due to the scarcity of organs [1]. Patient factors, including age, frailty, and cognitive status, can be used during the evaluation process to determine eligibility for transplantation but their use is not standardized thereby raising concerns about equity in patients’ access to transplantation. While practices may not be uniform across centers in the US, the ethical guidance of including frailty and cognitive impairment at KT evaluation should be consistent.

Adults aged 65 years or older comprised over 40% of patients with end-stage renal disease (ESRD), but only represented 20% of all KT recipients in the US [1]. Although older patients with ESRD who undergo KT double their life expectancy and report better health-related quality of life (HRQOL) than those who remain on hemodialysis (HD), referral for KT in older patients is only a fraction of that observed in younger patients [2–4], raising concerns about age-based inequities in access to transplantation. To better assess which patients would benefit from KT, clinical teams are measuring physiologic and cognitive aging to help evaluate older ESRD patients amidst a shortage of deceased donor organs available for KT [5, 6].

Frailty and cognitive impairment are measures of physiologic and cognitive aging that are well-suited for assessing the eligibility of older adults undergoing KT evaluation, and are used by some clinicians to guide listing decisions [5–8]. Frailty is a clinical syndrome of decreased physiologic reserve and increased vulnerability to stressors, which is commonly measured using the physical frailty phenotype developed by Dr. Fried and colleagues in the field of organ transplantation [9]. The physical frailty phenotype measures five criteria including unintentional weight loss, exhaustion, low energy expenditure, low grip strength, and/or slowed walking speed [9]. As measured by the physical frailty phenotype, 50% of hemodialysis patients and 21% of KT candidates are frail [9, 10]. Among ESRD patients, frailty is associated with falls, hospitalizations, cognitive impairment, decreased HRQOL, and mortality as well as a lower chance of being waitlisted for KT [10–14]. However, frail patients also experience a restoration of kidney function and improvement in frailty following transplantation [15, 16].

Cognitive impairment is characterized by impairments in memory, language, and/or judgement. It refers to cognitive decline that is above and beyond what is expected in normal cognitive aging but not having progressed to dementia. It is commonly measured using the Montreal Cognitive Assessment (MoCA) or the Mini Mental State Exam (MMSE). Around 55% of KT candidates are cognitively impaired as defined using the MoCA, with the majority of candidates having mild-moderate cognitive impairment as opposed to severe or no cognitive impairment [17]. Cognitively impaired candidates can have a hard time completing the transplant evaluation and adhering to their medical treatment [17, 18]. Adverse outcomes such as higher risk of graft failure and mortality are associated with cognitive impairment, yet cognitive function is also shown to improve following KT [12, 19]. Although cognitive impairment is common in KT candidates, these candidates have a 25% lower chance of being waitlisted for KT than non-cognitively impaired candidates [17].

Despite the increasing use of frailty and cognitive impairment to evaluate patients’ eligibility for transplantation, ethical guidance is lacking on how to appropriately use these measures when making waitlisting decisions. Current ethical guidance for the allocation of deceased donor organs stems from the Organ Procurement and Transplantation Network (OPTN) [20]. The OPTN aims to create a fair and efficient system of organ allocation by balancing the ethical principles of utility (maximizing benefits from transplantation), equity (equal access to transplantation), and respect for persons [20]. However, these guidelines do not explicitly address how to balance these principles when deciding whether or not to list a patient who is frail or has cognitive impairment. Consequently, inequities persist. The first step in developing ethical guidelines is to develop a clinical consensus among experts on whether and how to incorporate frailty and cognitive function in the KT evaluation.

In this study we sought clinical consensus on the use of frailty and cognitive impairment during KT evaluation from a group of experts during a Delphi panel.

## Methods

### Study design

We used a modified, virtual Delphi method, a widely used anonymous group survey method for gathering data from an expert panel through multiple rounds of open-ended and structured questioning [21–23]. Expert Delphi panels have been widely used in clinical medicine and have shown to be an effective and reliable method to develop consensus when conducting surveys with a small sample size given that the expert panel stems from a clearly defined knowledge area [24]. There is currently no set standard for a sufficient number of Delphi participants although sample sizes range from 15 to over a 100 [24, 25]. In this study, we conducted two rounds of data collection through iterative surveys distributed online using Qualtrics (Qualtrics LLC, Salt Lake City, UT). This study was deemed exempt by the Johns Hopkins School of Medicine Institutional Review Board (IRB00260939). Completion of survey served as consent to participate in the research study, as outlined in the beginning of the online survey.

### Study population

Eligible participants were clinicians who were familiar with frailty and cognitive function measures and cared for ESRD patients in the United States, namely, transplant surgeons, nephrologists, transplant coordinators, geriatricians, transplant infectious disease specialists as well as advanced practice providers. We sampled these experts from our existing databases that have been used for a previously published national survey, including clinicians from different transplant centers across the United States [6]. Experts were recruited via email by the principal investigator (MMD).

### Data collection

Round 1: The first-round survey instrument was developed based on a literature review surrounding the ethics of using age, frailty, and cognitive impairment to determine patient eligibility for kidney transplantation. Round 1 question items included statements and lists of factors for experts to rate on a 5-point Likert scale as well as open-ended questions about other factors to consider in transplant evaluation. Additionally, experts were asked an open-text question regarding how they balance of utility and justice in organ allocation. Experts were given 6 weeks to complete the survey. The survey instrument for round 1 is presented in Appendix 1.

Round 2: Three weeks following the end of round 1, the round 2 survey was finalized, approved by the Johns Hopkins School of Medicine IRB, and distributed to experts who had participated in the prior round. Experts were provided with a list of factors from round 1 to consider in evaluating patients for transplantation in general, as well as under scenarios related to graft survival, age, frailty, or cognitive impairment. If the factors were rated in round 1 as ‘very important’ or ‘important’ or selected by at least 25% of experts as factors that would cause them to list a patient for KT, then the factor was included in round 2. (Figure S1) Round 2 also included new factors that were added by at least two experts in round 1. We did not include factors in the round 2 that 100% of experts rated as ‘very important’ or ‘important’ in round 1. For factors that were previously rated in round 1, we provided experts with the percentage of experts who rated each factor as ‘very important’ or ‘important’. Experts were asked to rate the importance of these factors on a 5-point Likert scale. The survey instrument for round 2 is presented in Appendix 2.

### Analysis

Consensus was defined as > 80% agreement. Descriptive statistics were calculated using Stata 16.1 (StataCorp, College Station, Texas). Through discussion, two members of the study team (PS, SER) identified common themes among responses to the single open-text question.

## Results

### Study population

For this study, 27 experts participated in round 1, representing 18 centers (92% academic centers) and 9 of the 11 United Network for Organ Sharing (UNOS) regions. Most round 1 experts (67%) were female. Experts included nephrologists (41%), transplant surgeons (22%), geriatricians (11%), nurses, nurse practitioners, or physician assistants (11%), transplant coordinators (7%), and transplant infectious disease specialists (7%) (Table 1). Most experts (73%) had worked as a transplant clinician for ≥5 years. Of the 27 experts participating in round 1, 21 (78%) also participated in round 2.

**Table 1:** Characteristics of the geriatric transplant experts who completed Round 1

Factor	Response	Frequency (%)
N		27
Gender	Female	18 (67%)
Clinical Role	Transplant Nephrologist	7 (26%)
	Transplant Surgeon	6 (22%)
	General Nephrologist	4 (15%)
	Nurse/PA/NP	3 (11%)
	Geriatrician	3 (11%)
	Transplant Infectious Diseases Specialist	2 (7%)
	Transplant Coordinator/Nurse Administrative Manager	2 (7%)
Years worked as a transplant clinician	15–24 years	5 (19%)
	5–14 years	14 (54%)
	< 5 years	7 (27%)
United Network for Organ Sharing (UNOS) Region	Region 2	5 (19%)
	Region 3	1 (4%)
	Region 4	2 (8%)
	Region 5	2 (8%)
	Region 7	8 (31%)
	Region 8	2 (8%)
	Region 9	2 (8%)
	Region 10	3 (12%)
	Region 11	1 (4%)
Self-reported familiarity with literature on frailty in kidney transplantation	Very familiar	9 (33%)
	Familiar	16 (59%)
	Unfamiliar	2 (7%)
Frequency of measurement of frailty during transplant evaluation	Always	11 (42%)
	Sometimes	1 (4%)
	Never	4 (15%)
	Not sure	10 (38%)
Age used to determine if patients’ frailty status should be measured	Yes	8 (40%)
	No	12 (60%)
Self-reported familiarity with literature on cognitive impairment in kidney transplantation	Very familiar	5 (19%)
	Familiar	12 (44%)
	Unfamiliar	10 (37%)
Frequency of measurement of cognitive impairment during transplant evaluation	Always	4 (16%)
	Sometimes	14 (56%)
	Never	2 (8%)
	Not sure	5 (20%)
Aged used to determine if patients’ cognitive status should be measured	Yes	7 (39%)
	No	11 (61%)

### Knowledge and use of frailty and cognitive impairment in kidney transplantation

Most (93%) experts reported being ‘very familiar’ or ‘familiar’ with the published literature on frailty in transplant patients, and 42% stated that frailty was ‘always’ measured at their center during transplant evaluation (Table 1). Thirty-eight percent of experts reported that frailty was ‘sometimes’ measured at their center during transplant evaluation, depending on patients’ age, comorbidity criteria, or by physician discretion, such as perceived frailty on clinical exam (Table 1). Experts used different frailty assessment tools to ascertain frailty status.

Sixty-three percent of experts reported being ‘very familiar’ or ‘familiar’ with the published literature on cognitive function in transplant patients. Only 16% of experts reported that cognitive function was ‘always’ measured at their center during transplant evaluation, while 56% measured cognitive function ‘sometimes’ at their center. In these cases, experts reported deciding to measure cognitive function based on risk identified by social worker, clinical concern, patient self-report, or patient history. Forty percent of experts reported using the Montreal Cognitive Assessment (MoCA) test, a validated cognitive screening tool, as one of the cognitive assessment tools, followed by 17% who used Mini Mental State Exam (MMSE), and 17% who used self-reported (or proxy reported) cognitive function.

## Delphi results

### General factors that were considered during transplant evaluation

In round 1, at least 80% of the experts rated the following factors as ‘very important’ or ‘important’: frailty, cognitive impairment, cardiovascular disease, patient adherence/compliance to treatment, social support, psychosocial issues such as substance abuse, surgical complexity such as vascular disease, improved quality of life (QOL) or benefit from transplant, cancer/infectious history, patient preference for transplant, age, lack of access to other options (e.g., being ineligible for dialysis or not having a living donor), and the number of comorbidities (Table 2). After both rounds of surveys, 100% of experts rated frailty, cognitive impairment, cardiovascular disease, patient adherence/compliance to treatment, social support, and psychosocial issues (e.g., substance abuse) as ‘very important’ or ‘important’ to consider in transplant evaluation. Experts also reached consensus after round 2 that the following factors were ‘very important’ or ‘important’ to consider before listing a patient for KT: surgical complexity (e.g. vascular disease), improved QOL or benefit from transplant, cancer/infectious history, patient preferences for transplant, age, lack of access to other options such as dialysis or living donor, and number of comorbidities (Table 2). There was no consensus on the following factors in either round 1 or round 2: obesity/BMI, diabetes, tolerance to dialysis, history of previous transplant, symptoms associated with kidney disease, and time on dialysis (Table 2).

**Table 2:** Factors to consider for transplant evaluation

	Round 1		Round 2	
Factors	Rated important or very important, n	%	Rated important or very important, n	%
Frailty	25	100%	*	*
Cognitive Impairment	25	100%	*	*
Cardiovascular disease	24	96%	21	100%
Patient adherence/compliance to treatment	**	**	21	100%
Social support	22	88%	21	100%
Psychosocial issues (e.g. substance abuse)	**	**	21	100%
Surgical complexity (e.g. vascular disease)	**	**	20	95%
Improved QOL or benefit from transplant	**	**	19	90%
Cancer/Infectious history	**	**	19	90%
Patient preference for transplant	**	**	18	86%
Age	20	80%	17	81%
Lack of access to other options (dialysis or living donor)	**	**	18	86%
Number of comorbidities	**	**	17	81%
Obesity/BMI	17	68%	16	76%
Diabetes	17	68%	12	57%
Tolerance to dialysis	12	48%	9	43%
History of previous transplant	11	44%	8	38%
Symptoms associated with kidney disease	10	40%	8	38%
Time on dialysis	9	36%	7	33%

*Experts were not asked to rate frailty and cognitive impairment in round 2 as they were in 100% agreement in the first round

** New factor that was suggested by ≥2 experts in round 1, then subsequently included in round 2

### Considerations of waitlisting a patient unlikely to outlive the graft

Seventy-two percent of experts ‘strongly agreed’ or ‘agreed’ that it is appropriate to waitlist a patient who is unlikely to outlive the graft for transplantation. In round 1, 76% of experts and 72% of experts selected health-related quality of life (HRQOL) and younger age, respectively, as factors that would cause them to waitlist a patient who is unlikely to outlive the graft (Table 3; Table S1). In round 2, there was consensus that HRQOL and younger age are ‘very important’ or ‘important’ factors that would cause them to list a KT candidate that is unlikely to outlive the graft (Table 3). Experts did not reach consensus on the importance of benefit of transplantation over dialysis to the patient, symptoms on dialysis, social support, or dependence in activities of daily living for patients who are unlikely to outlive the graft in either round 1 or 2 (Table 3; Table S1).

**Table 3:** Factors to consider for transplant evaluation of a patient who is unlikely to outlive the graft, 65 years or older, frail, or cognitively impaired

	% of experts who rated the factor after two rounds as very important or important to consider when listing a KT candidate for transplantation who is:			
Factor	Unlikely to outlive the graft	Aged ≥65 years	Frail	Cognitively impaired
Health Related Quality of Life (HRQOL)	**95%**	**95%**	**85%**	**95%**
Social Support	50%	**100%**	**95%**	**100%**
Medication adherence	***	**100%**	***	**95%**
Low or no frailty*	***	**95%**	***	***
Younger Age	**85%**	*	**90%**	70%
Good functional status*	***	**100%**	***	***
Benefit over dialysis to patient*	75%	***	***	***
Low comorbidity burden*	***	**80%**	***	***
No medical contraindications*	***	**95%**	**90%**	***
Symptoms on dialysis	60%	***	35%	***
Dependence in activities of daily living	40%	35%	**	**
Race/Ethnicity	**	**	**	**
Sex	**	**	**	**
Frailty could be reversible or improved (from transplant or prehab)	***	***	**95%**	***
Likelihood for success or benefit from surgery	***	***	**95%**	***
Degree of cognitive impairment (e.g., mild vs. dementia)	***	***	***	**95%**
Cognitive impairment related to ESRD/HD symptoms and not a primary neurocognitive disorder	***	***	***	75%

*New factor that was suggested by ≥2 experts in round 1, then subsequently included in round 2

** Experts were not asked to rate factors in round 2 that were rated very important/important by < 25% in round 1

*** Factor not relevant to this patient

### Considerations of listing a patient who is 65 years or older

**Fig. 1:**
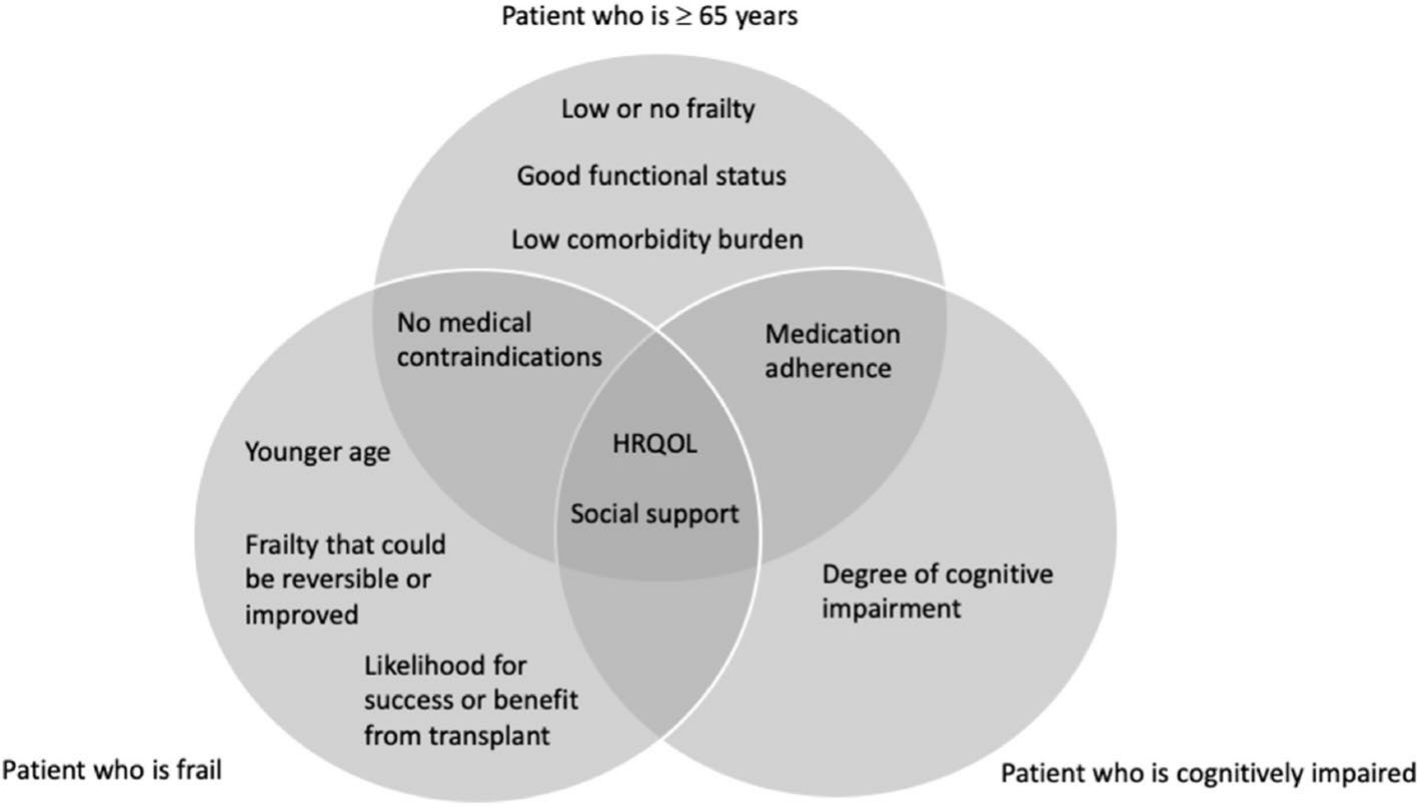
Venn diagram of factors that were considered very important or important to consider before listing patients who are 65 years or older, frail, or cognitively impaired, for a KT

Ninety-six percent of the experts ‘strongly agreed’ or ‘agreed’ that it is appropriate to list a patient who is 65 years or older for transplantation, while one expert was undecided, and none disagreed with the statement. In round 1, good functional status, good social support, and medication adherence were selected as factors that would cause them to list a patient who is 65 years or older by 76, 72, and 65% of experts, respectively (Table 3; Table S2). After two rounds of the Delphi panel, at least 80% rated the following factors as ‘very important’ or ‘important’ to consider when evaluating patients who are 65 or older: good social support, medication adherence, good functional status, HRQOL, low or no frailty, no medical contraindications, and low comorbidity burden (Table 3; Fig. 1). Experts did not reach consensus on the importance of symptoms of dialysis and dependence in activities of daily living for patients who are 65 years or older in either round.

### Considerations of waitlisting a patient who is frail

Seventy-two percent of the experts ‘strongly agreed’ or ‘agreed’ that it is appropriate to waitlist a transplant candidate who is frail. In round 1, 64% of participants selected HRQOL and younger age, and 52% selected social support as factors that would cause them to list a frail patient for KT (Table S3). After round 2, at least 80% of participants rated the following factors that would cause them to list a frail patient as ‘very important’ or ‘important’: social support, frailty that could be reversible or improved from either transplant or prehabilitation, likelihood for success or benefit from surgery, younger age, no medical contraindications, and HRQOL (Table 3; Fig. 1). Experts did not reach consensus on symptoms of dialysis in either round (Table 3; Table S3).

### Considerations of waitlisting a patient who is cognitively impaired

Sixty-eight percent of the experts ‘strongly agreed’ or ‘agreed’ that it is appropriate to list a transplant candidate who is cognitively impaired. 72 and 60% of experts selected social support and HRQOL, respectively, as factors that would cause them to list a cognitively impaired patient for KT (Table S4). After both rounds of surveys, 100% of experts considered social support a ‘very important’ or ‘important’ factor that would cause them to list a cognitively impaired patients for transplantation (Table 3; Fig. 1). In addition, 95% of the experts rated HRQOL, the degree of cognitive impairment, and medication adherence as ‘very important’ or ‘important’ factors that would cause them to list a cognitively impaired patient for transplantation. (Table 3; Fig. 1). Experts did not reach consensus for the following factors in either round: younger age or cognitive impairment related to ESRD/dialysis symptoms that is not a primary neurocognitive disorder. (Table 3; Table S4).

### Perspectives on utility in organ allocation

When experts were asked an open-text question about whether it was ‘fair’ to allocate organs to patients who will live for a long time with the organ, thus giving younger, physically healthy, and cognitively intact people an advantage in gaining access to transplants, many experts did not believe that this was a fair approach to organ allocation.

Not a fair approach. If patients can live longer and have better quality of life with a transplant than [sic] they deserve a chance at transplant.

Clinician A

These experts believed that discriminating solely based on patients’ age, frailty status, or cognitive status was unfair:

Discriminating solely based on age, frailty status, disability, cognition, etc. is unfair to older adults and perpetuates ageism and ableism.

Clinician B

Experts also expressed that the length of life should not be the only consideration as it puts older patients at a disadvantage; instead, quality of life should also be considered:

In my opinion, prolonging survival is only one of the benefits of transplantation relative to dialysis. At our program, we factor in potential benefits such as the freedom afforded by transplant and quality of life when we weigh the risks and benefits of transplantation with our patients.

Clinician C

However, most experts agreed that in the context of limited organs, longevity matching to prioritize healthiest organs for the healthiest candidates was a fair approach that could still allow older, frail, and cognitively impaired candidates to receive benefit from available organs, perhaps even from non-standard or marginal organs:

Ideally expected recipient and kidney survival should be matched. This is the most fair long-term [approach] to avoid wasting transplant organs and decreasing the likelihood younger patients need to return to the list. Such an approach could still have good access to older candidates to kidneys that would provide life improvement benefit.

Clinician D

A few experts called for structural changes and improvements in listing and allocation practices across different transplant centers:

It is important to have consistent listing practices across transplant centers to have truly fair organ allocation.

Clinician E

In principle, I agree with the idea of longevity matching as a way to provide the highest quality kidneys to those who are most likely to need those organs for the longest time. However, our current paradigm to predict longevity doesn't account for modifiable causes of vulnerability. A fairer process would identify and address these root causes.

Clinician C

Overall, experts were against denying patients transplantation based solely on the patients’ expected survival as it would be unfair to older, frail, or cognitively impaired patients. Experts expressed that matching expected recipient and organ survival was key to balancing maximized use of available organs while enabling everyone who could benefit from transplantation to have access to KT. Furthermore, experts believed that the benefit to patients should not be measured solely based on prolonged survival but also based on improvement in HRQOL.

## Discussion

To our knowledge this is the first study to obtain clinical consensus among experts on ethical issues regarding access to transplantation for frail and cognitively impaired candidates. Our panel of experts believed it was unfair to allocate organs based only on patients’ age, frailty status, cognitive status, or their likelihood of outliving the graft. However, experts agreed that when deciding whether to waitlist an older, frail, or cognitively impaired patient, other factors, like social support, are important to consider.

Experts believed that denying access to transplantation based solely on patients’ expected survival was unfair and solely using factors such as age, frailty status, or cognitive status was “discriminatory” and “unfair”. Instead, experts believed that providing access to transplant to those who could benefit from it, in terms of life expectancy and improvements in quality of life, was the most ethically acceptable approach to allocation. Given the organ shortage, experts thought that longevity matching was a robust solution to resolving tensions between commitments to maximize the utility of available organs and treating all patients who would benefit from transplantation, including older, frail, or cognitively impaired adults, fairly. These findings reflect current clinical knowledge that older adults do benefit from transplantation compared to remaining on the waitlist, even in age-matched KT recipients and high-Kidney Donor Profile Index (KDPI) recipients [26, 27]. Furthermore, they align with current OPTN ethical guidelines that aim to balance utility and equity in organ allocation [3, 20, 26] as well as the 2014 policy change in the national kidney allocation system (KAS) that aimed to eliminate longevity mismatch, among other strategies to increase both equity and efficiency in organ allocation [28]. While the new KAS has increased kidney utility by reducing the gap between expected patient and graft survival, it has not increased equity in kidney allocation [28].

Although most experts believed that patients should not be denied a place on the waitlist based solely on patient or graft survival, experts did reach consensus on multiple factors that are important to consider when evaluating general KT candidates: frailty, cognitive impairment, cardiovascular disease, patient adherence or compliance to treatment, social support, and psychosocial issues. These findings are consistent with current waitlisting practices and previous studies that found that transplant providers were less likely to recommend patients for transplantation if they had comorbidities, had no social support, were non-adherent, and were older [29, 30]. By contrast, experts did not identify race, ethnicity, socioeconomic status, or sex as important considerations in KT evaluation, despite waitlisting trends demonstrating lower access to KT among patients who are racial and ethnic minorities, women, or belong to a lower socioeconomic status [31–36]. It is important to note that even though providers did not explicitly list socioeconomic status as an important factor, social support and socioeconomic status are often related and thus decisions made during transplant evaluations could be unfavorable to patients of low socioeconomic status [30, 37].

Lack of comorbidities and no medical contraindications were considered important by experts when evaluating patients who were 65 years or older. Previous studies have also highlighted similar considerations of comorbidity burden, when evaluating older adults [38, 39]. Experts reached consensus that HRQOL is an important consideration among older patients, frail patients, cognitively impaired patients, and patients unlikely to outlive the graft. This result aligns with current evidence that frail and older KT recipients experience an improved quality of life after transplantation [14, 40–42]. There was also consensus that that good social support is important to consider when evaluating KT candidates who are over 65 years of age, frail, or cognitively impaired. While previous research has also demonstrated that clinicians strongly favor candidates with good social support, there is a lack of evidence regarding whether social support provides any clinical utility, and in fact may increase inequities in transplantation [43, 44].

These findings suggest that HRQOL and social support are common factors to consider in older patients, even if they are not frail or cognitively impaired [12, 45].

When evaluating frail patients for KT, there was consensus that younger age, reversibility of frailty following a transplant or prehabilitation, and likelihood for success or benefit from transplant are also important factors. This finding is consistent with previous research showing that clinicians believed pre-habilitation would help ESRD patients and make them less frail [5]. Additionally, experts considered no medical contraindications an important factor that would cause them to list an older or frail patient for KT.

Among cognitively impaired patients, experts reached consensus that the degree of cognitive impairment, such as whether a patient had dementia versus ESRD related and mediated cognitive impairment, was an important factor. This finding is relevant for clinical practice as there is evidence suggesting that KT leads to improved cognitive function [12, 46]. There was also consensus that medication adherence is an important consideration for cognitively impaired or older patients, reflecting concerns raised in previous studies [46].

Our study had limitations. First, this study consisted of a small sample (< 30) of clinicians who are experts in the field of kidney transplantation. However, the Delphi method is designed to develop consensus among a small group of experts and our panel size is consistent with previously published Delphi studies [5, 25, 47]. Despite the small sample size, the reported knowledge of the experts on frailty and cognitive impairment in kidney transplantation was high. 93% of experts reported high familiarity with the literature on frailty. Fewer experts reported familiarity with literature on cognitive function than frailty, thus potentially having an incomplete understanding of cognitive function in ESRD and KT, but the majority (63%) of experts still reported high familiarity with literature on cognitive impairment. The high levels of familiarity suggest that the experts in our study were able to provide informed responses to our questions, adding to the strength of our study. While the Delphi technique does not require or assure representativeness [25], our expert panel was diverse geographically with experts representing 9 out of 11 UNOS regions, as well as in the clinical roles represented. Although the majority were either nephrologists or transplant surgeons, our panel also included geriatricians, transplant infectious disease specialists, advanced practice professionals, and transplant coordinators/nurse administrative managers.

Current waitlisting practices vary widely between transplant centers, and recommendations for waitlisting consist of broadly defined, and largely subjective measures [48–50]. Even more inconsistent than the general measures for waitlisting are the guidelines and tools employed to evaluate older adults that use constructs like frailty and cognitive impairment across the United States [6, 50]. As clinicians are more commonly using measures of aging such as frailty and cognitive impairment in the evaluation of KT candidates, consistent ethics guidance to ensure appropriate and fair use of these constructs across transplant centers is critically needed [5, 6]. Organizations like the American Society for Transplantation (AST) can play an important role in providing national guidelines to measure and utilize frailty and cognitive impairment during transplant evaluation more equitably.

## Conclusions

To prevent inequities in access to transplantation and aid transplant programs in the evaluation of frail and cognitively impaired candidates, it is essential to develop a set of consistent ethical guidelines regarding how to balance utility and equity in the evaluation of frail and cognitively impaired candidates. The clinical consensus developed in this study is an important step toward developing this ethical guidance. This is an important first step in developing national ethical guidelines to help balance issues of utility and justice when considering frail and cognitively impaired candidates. This study is the first to consider the ethical considerations of using frailty and cognitive function in clinical practice and can help inform other subspecialties that seek to measure these constructs in their practice.

## Supplementary Information

**Additional file 1.**

## Data Availability

The datasets used and/or analyzed during the current study are available from the corresponding author on reasonable request. We are unable to deposit the data in a publicly available source because there are ongoing NIH studies using this data.

## Abbreviations

BMI: Body Mass Index
ESRD: End-stage Renal Disease
HRQOL: Health-related Quality of Life
IRB: Institutional Review Board
KDPI: Kidney Donor Profile Index
KT: Kidney Transplantation
MMSE: Mini Mental State Exam
MoCA: Montreal Cognitive Assessment
OPTN: Organ Procurement and Transplantation Network
UNOS: United Network for Organ Sharing
