# Supplementary material for: Defining the ethical considerations surrounding kidney transplantation for frail and cognitively impaired patients: a Delphi study of geriatric transplant experts
Source: BMC Geriatr. 2022 Jul 8;22:566. doi: 10.1186/s12877-022-03209-x (PMC9264705; doi:10.1186/s12877-022-03209-x)
Supplement: Supplementary file 1 — Additional file 1. [file 12877_2022_3209_MOESM1_ESM.pdf]

Table S1. Factors to consider for transplant evaluation of a patient who is unlikely to outlive the graft.

	Round 1		Round 2	
**Factors**	**Factor that would cause participant to list patient, n**	**%**	**Rated very important or important, n**	**%**
Health-related quality of life	19	76%	19	95%
Younger age	18	72%	17	85%
Benefit over dialysis to patient	*	*	15	75%
Symptoms on dialysis	13	52%	12	60%
Social support	9	36%	10	50%
Dependence in activities of daily living	7	28%	8	40%
Medication Adherence	6	24%	**	**
Race/Ethnicity	1	4%	**	**
Sex	0	0	**	**

*New factor that was suggested by ≥2 experts in round 1, then subsequently included in round 2

** Experts were not asked to rate factors in round 2 that were rated very important/important by <25% in round 1

Table S2. Factors to consider for transplant evaluation of a patient who is 65 years or older.

Factors	Round 1<br>Factor that would cause participant to list patient, n	%	Round 2<br>Rated very important or important, n	%
Good social support	18	72%	20	100%
Medication adherence	16	64%	20	100%
Good functional status	*	*	20	100%
Health-related quality of life	19	76%	19	95%
Low or no frailty	*	*	19	95%
No medical contraindications	*	*	19	95%
Low comorbidity burden	*	*	16	80%
Symptoms on dialysis	11	44%	10	50%
Dependence in activities of daily living	7	28%	7	35%
Race/Ethnicity	1	4%	**	**
Sex	0	0	**	**

*New factor that was suggested by ≥2 experts in round 1, then subsequently included in round 2

** Experts were not asked to rate factors in round 2 that were rated very important/important by <25% in round 1

Table S3. Factors to consider for transplant evaluation of a patient who is frail.

	Round 1		Round 2	
**Factor**	**Factor that would cause participant to list patient, n**	**%**	**Rated very important or important, n**	**%**
Social support	13	52%	19	95%
Frailty could be reversible or improved (from transplant or prehab)	*	*	19	95%
Likelihood for success or benefit from surgery	*	*	19	95%
Younger age	16	64%	18	90%
No medical contraindications	*	*	18	90%
Health-related quality of life	16	64%	17	85%
Symptoms on dialysis	12	48%	7	35%
Dependence in activities of daily living	6	24%	**	**
Race/Ethnicity	0	0	**	**
Sex	0	0	**	**

*New factor that was suggested by ≥2 experts in round 1, then subsequently included in round 2

** Experts were not asked to rate factors in round 2 that were rated very important/important by <25% in round 1

Table S4. Factors to consider for transplant evaluation of a patient who is cognitively impaired.

	Round 1		Round 2	
**Factor**	**Factor that would cause participant to list patient, n**	**%**	**Rated very important or important, n**	**%**
Social support	18	72%	20	100%
Health-related quality of life	15	60%	19	95%
Degree of cognitive impairment (e.g. mild vs. dementia)	*	*	19	95%
Medication adherence	*	*	19	95%
Cognitive impairment related to ESRD/HD symptoms and not a primary neurocognitive disorder	*	*	15	75%
Younger age	10	40%	14	70%
Dependence in activities of daily living	3	12%	**	**
Race/Ethnicity	0	0	**	**
Sex	0	0	**	**

*New factor that was suggested by ≥2 experts in round 1, then subsequently included in round 2

** Experts were not asked to rate factors in round 2 that were rated very important/important by <25% in round 1

Figure S1. Flowchart of factors introduced and removed at each round. Experts were asked to report which factors would cause them to list a patient for each scenario in round 1 and in round 2 were asked to rate the importance of each factor. The different scenarios were a) General transplant evaluation; b) Patient who is unlikely to outlive the graft; c) Patient who is 65 years or older; d) Patient who is frail; e) Patient who is cognitively impaired.

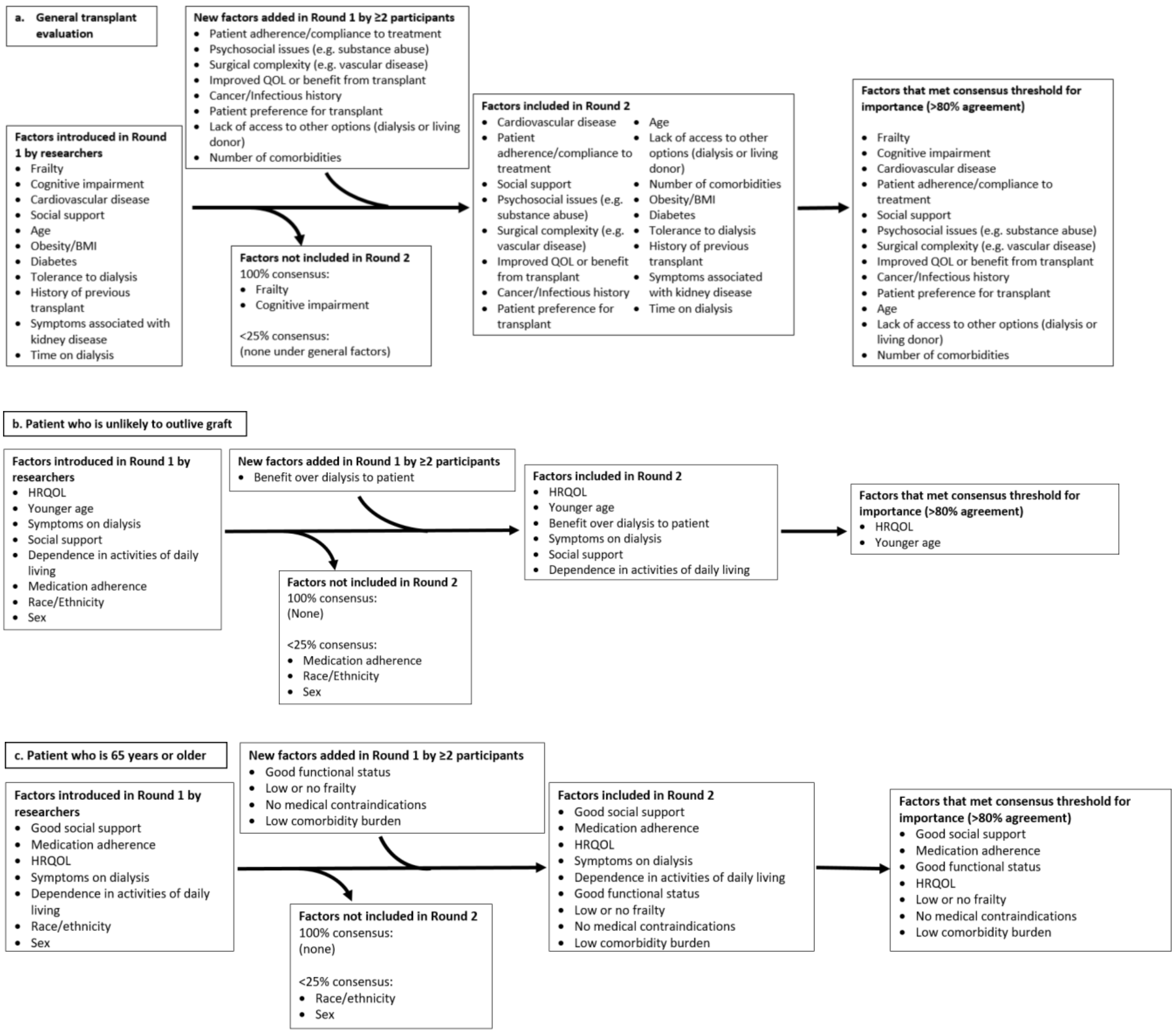

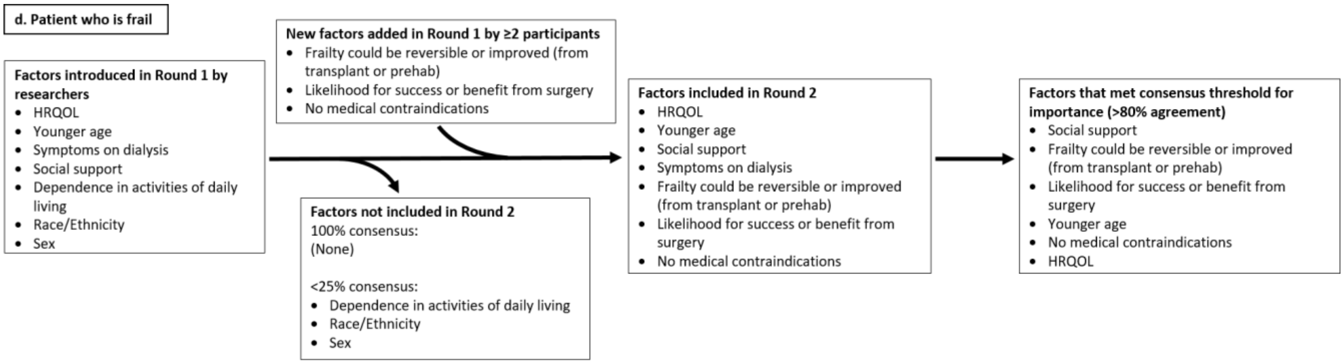

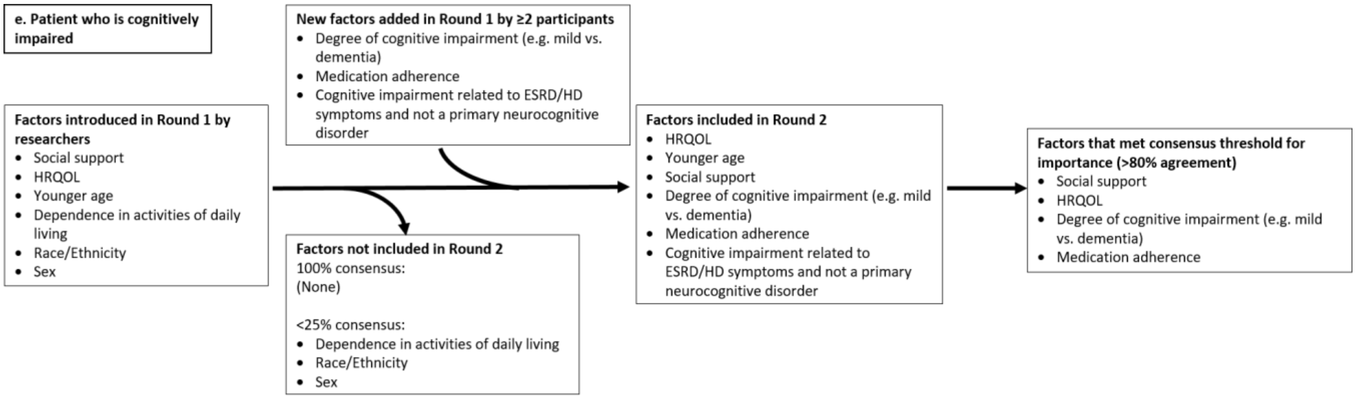

Appendix 1. Round 1 survey instrument.

Delphi Clinician Survey Round 1

Throughout this Delphi panel, we will send you up to four surveys about ethical issues in the transplantation of older, frail, or cognitively impaired adults.

This first survey aims to assess clinicians' perspectives on if and when frailty and cognitive function should be used as candidate eligibility criteria for kidney transplantation.

Your completion of this survey will serve as your consent to participate in this research study.

Survey Questions	Response Format	Response Options
**Participant Background**		
1. What is your clinical role?	Select one	a. Transplant Nephrologist<br>b. Transplant Surgeon<br>c. Transplant Coordinator<br>d. Nurse/PA/NP<br>e. Pharmacist<br>f. Social Worker<br>g. Other (open text)

2. What is your center's OPTN code (e.g., MDJH)?	Dropdown	4 digit OPTN Code
3. How many years have you been practicing as a transplant clinician?	Select one	a. <5 years<br>b. 5-14 years<br>c. 15-24 years<br>d. ≥25 years
4. What is your sex?	Select one	a. Female<br>b. Male
5. What is your gender?	Select one	a. Woman<br>b. Man<br>c. Transgender<br>d. Non-binary/non-conforming<br>e. Prefer not to say
6. How familiar are you with the published literature on frailty among transplant candidates and recipients?	Select one	a. Very familiar<br>b. Familiar<br>c. Unfamiliar<br>d. Very unfamiliar
7. How familiar are you with the published literature on cognitive function among transplant candidates and recipients?	Select one	a. Very familiar<br>b. Familiar<br>c. Unfamiliar<br>d. Very unfamiliar
Frailty and Cognitive Function Practices		
8. How often do you measure frailty at your center during transplant evaluation?	Multiple choice; free text if (b)	a. Always<br>b. Sometimes (Please explain)<br>c. Never<br>d. Not sure
9. If Always or Sometimes to Q8: What frailty assessment tool(s) do you currently use? Mark all that apply.	Mark all that apply; free text if (v)	a. Timed walk (i.e. 6-minute walk test)<br>b. Body mass index to quantify wasting<br>c. Functional status (i.e. KDQOL, SF-36, or PCS)<br>d. Stair climbing assessment<br>e. Timed up and go<br>f. Number of hospitalizations in the previous year<br>g. Distance walk (i.e. 10-meter walk test)<br>h. Fried frailty phenotype

		i. Montreal Cognitive Assessment (MoCA)<br>j. Level of sarcopenia<br>k. Cognitive function (i.e. 3MS, MMSE, or Trail Making Test)<br>l. Short physical performance battery<br>m. Geriatric assessment<br>n. Frailty/Vigor assessment<br>o. Deficit Index (DAI, also called Frailty Index)<br>p. Gill Frailty Measure<br>q. Brief Frailty Instrument<br>r. Vulnerable Elders Survey<br>s. FRAIL scale<br>t. Rockwood clinical frailty scale<br>u. Winograd screening instrument<br>v. Other (please describe)<br>w. Don't know
10. If yes to Q8: Do you use age to determine when to start measuring patients' frailty status?	Select one; free text if (a)	a. Yes: At what age do you start measuring patients' frailty status?<br>b. No
11. How often do you measure cognitive function at your center during transplant evaluation?	Select one; free text if (b)	a. Always<br>b. Sometimes (please explain)<br>c. Never<br>d. Not sure
12. If Always or Sometimes to Q11: What cognitive assessment tool(s) do you currently use? Mark all that apply.	Select one; free text if (f)	a. Montreal Cognitive Assessment (MoCA)<br>b. Mini Mental State Exam (MMSE)<br>c. Modified Mini Mental State Test (3MS)<br>d. Trail making test A or B<br>e. Self-reported (or proxy reported) cognitive function<br>f. Other (Please describe)<br>e. Don't know

13. If yes to Q11: Do you use age to determine when to start measuring patients' cognitive status?	Select one; free text if (a)	a. Yes: At what age do you start measuring patients' cognitive status?<br>b. No
**Factors for clinical decision making**<br>In your opinion, please rate the following patient factors in terms of importance for deciding whether or not to add a patient to the kidney transplant waitlist.		
Age	Select one	a. Very Unimportant<br>b. Unimportant<br>c. No opinion<br>d. Important<br>e. Very Important<br>f. Not applicable
Cognitive impairment	Select one	a. Very Unimportant<br>b. Unimportant<br>c. No opinion<br>d. Important<br>e. Very Important<br>f. Not applicable
Cardiovascular disease	Select one	a. Very Unimportant<br>b. Unimportant<br>c. No opinion<br>d. Important<br>e. Very Important<br>f. Not applicable
Diabetes	Select one	a. Very Unimportant<br>b. Unimportant<br>c. No opinion<br>d. Important<br>e. Very Important<br>f. Not applicable
Frailty	Select one	a. Very Unimportant<br>b. Unimportant<br>c. No opinion<br>d. Important<br>e. Very Important<br>f. Not applicable
Obesity/Body Mass Index (e.g. $\geq 30 kg/m^2$)	Select one	a. Very Unimportant<br>b. Unimportant<br>c. No opinion<br>d. Important<br>e. Very Important<br>f. Not applicable
Social support	Select one	a. Very Unimportant<br>b. Unimportant<br>c. No opinion

		d. Important<br>e. Very Important<br>f. Not applicable
Tolerance to dialysis	Select one	a. Very Unimportant<br>b. Unimportant<br>c. No opinion<br>d. Important<br>e. Very Important<br>f. Not applicable
Time on dialysis	Select one	a. Very Unimportant<br>b. Unimportant<br>c. No opinion<br>d. Important<br>e. Very Important<br>b. Not applicable
History of previous transplant	Select one	a. Very Unimportant<br>b. Unimportant<br>c. No opinion<br>d. Important<br>e. Very Important<br>f. Not applicable
Symptoms associated with end stage kidney disease	Select one	a. Very Unimportant<br>b. Unimportant<br>c. No opinion<br>d. Important<br>e. Very Important<br>Not applicable
14. What other factors, not listed above, are important for deciding whether or not to add a patient to the kidney transplant waitlist?	Open text	

Ethical Considerations

As you may be aware, only about a quarter of patients on the kidney transplant waiting list received a transplant in 2018 and only about half of deceased donor transplant recipients survive for 10 or more years.

Patients who are frail and/or cognitively impaired generally have less access to transplantation, and have worse post-transplant outcomes than their non-frail or non-cognitively impaired peers. However, frail and/or cognitively impaired patients do benefit from transplantation, including improvements in: functional status, health-related quality of life, and cognitive function. Further, frailty and cognitive impairment are not specific to older patients. Younger patients with kidney failure also experience a high burden of frailty and cognitive impairment.

We’re interested in which factors should influence who has access to the transplant waitlist.

15. How much do you agree with the following statement?<br><br>It is appropriate to list a patient who is unlikely to outlive the graft.	Select one	a. Strongly Agree<br>b. Agree<br>c. Undecided<br>d. Disagree<br>e. Strongly Disagree
16. What factors would cause you to list a patient who is unlikely to outlive a graft for a transplant? [mark all that apply]	Mark all that apply; free text if (i) or (j)	a. Younger age<br>b. Race/Ethnicity<br>c. Sex<br>d. Social support<br>e. Medication adherence<br>f. Symptoms on dialysis<br>g. Health-related quality of life<br>h. Dependence in activities of daily living<br>i. Other [free text]<br>j. I would never list a patient who is unlikely to outlive a graft for transplantation (please explain)
17. How much do you agree with the following statements?<br><br>It is appropriate to list a patient who is 65 years or older.	Select one	a. Strongly Agree<br>b. Agree<br>c. Undecided<br>d. Disagree<br>e. Strongly Disagree
18. What factors would cause you to list a patient who is 65 years or older for a transplant? [mark all that apply]	Mark all that apply; free text if (h) or (i)	a. Race/ethnicity<br>b. Sex<br>c. Good social support<br>d. Medication adherence<br>e. Symptoms on dialysis<br>f. Health-related quality of life<br>g. Dependence in activities of daily living<br>h. Other [free text]<br>i. I would never list a patient who is 65 years or older for transplantation (please explain)<br>b.
19. How much do you agree with the following statement?<br><br>It is appropriate to list a patient who is frail.	Select one	a. Strongly Agree<br>b. Agree<br>c. Undecided<br>d. Disagree<br>e. Strongly Disagree

20. What factors would cause you to list a frail patient for a transplant? [mark all that apply]	Mark all that apply; free text if (i) or (j)	a. Race/ethnicity<br>b. Sex<br>c. Social support<br>d. Younger age<br>e. Medication adherence<br>f. Symptoms on dialysis<br>g. Health-related quality of life<br>h. Dependence in activities of daily living<br>i. Other [free text]<br>j. I would never list a frail patient for a transplant (please explain)
21. How much do you agree with the following statement?<br><br>It is appropriate to list a patient who is cognitively impaired.	Select one	a. Strongly Agree<br>b. Agree<br>c. Undecided<br>d. Disagree<br>e. Strongly Disagree
22. What factors would cause you to list a cognitively impaired patient for a transplant? [mark all that apply]	Mark all that apply; free text if (i) or (j)	a. Race/ethnicity<br>b. Sex<br>c. Social support<br>d. Younger age<br>e. Medication adherence<br>f. Symptoms on dialysis<br>g. Health-related quality of life<br>h. Dependence in activities of daily living<br>i. Other [free text]<br>j. I would never list a cognitively impaired patient for a transplant (please explain)
23. Some clinicians believe that organs should be allocated to patients who will live for a long time with the organ. Doing so would give younger, physically healthy, and cognitively intact people an advantage. In your opinion, is this organ allocation approach fair? Why? Why not?	Free text	

Appendix 2. Round 2 survey instrument.

1. **100% of your peers rated cognitive impairment and frailty very important or important. Please rate the following factors to consider in terms of importance for kidney transplant evaluation in terms of importance:**

**Factor for kidney transplant evaluation**	**Average rating from your peers (out of 5)**	**% of your peers who rated this factor very important/important**	**Your new rating**
CVD	4.48	96%	1. Very Unimportant<br>2. Unimportant<br>3. No opinion<br>4. Important<br>5. Very Important<br>6. Not applicable
Social Support	4.4	88%	1. Very Unimportant<br>2. Unimportant<br>3. No opinion<br>4. Important<br>5. Very Important<br>6. Not applicable
Age	3.78	80%	1. Very Unimportant<br>2. Unimportant<br>3. No opinion<br>4. Important<br>5. Very Important<br>6. Not applicable
Diabetes	3.52	68%	1. Very Unimportant<br>2. Unimportant<br>3. No opinion<br>4. Important<br>5. Very Important<br>6. Not applicable
Obesity/BMI	3.48	68%	1. Very Unimportant<br>2. Unimportant<br>3. No opinion<br>4. Important<br>5. Very Important<br>6. Not applicable
Tolerance to dialysis	3.08	48%	1. Very Unimportant

			2. Unimportant<br>3. No opinion<br>4. Important<br>5. Very Important<br>6. Not applicable
History of previous transplant	3.08	44%	1. Very Unimportant<br>2. Unimportant<br>3. No opinion<br>4. Important<br>5. Very Important<br>6. Not applicable
Symptoms associated with end stage kidney disease	2.92	40%	1. Very Unimportant<br>2. Unimportant<br>3. No opinion<br>4. Important<br>5. Very Important<br>6. Not applicable
Time on dialysis	2.76	36%	1. Very Unimportant<br>2. Unimportant<br>3. No opinion<br>4. Important<br>5. Very Important<br>6. Not applicable

2. Your peers identified additional factors in the first round of this delphi study that may or may not be important to consider for kidney evaluation. **<u>In your opinion,</u>** please rate the following patient factors in terms of importance for deciding whether or not to add a patient to the kidney transplant waitlist.	
**Patient Factors**	
ii. Patient adherence/compliance	7. Very Unimportant<br>8. Unimportant<br>9. No opinion<br>10. Important<br>11. Very Important<br>12. Not applicable
iii. Surgical complexity (e.g. vascular disease)	1. Very Unimportant<br>2. Unimportant<br>3. No opinion<br>4. Important<br>5. Very Important<br>6. Not applicable
iv. Psychosocial issues (e.g. substance abuse)	1. Very Unimportant<br>2. Unimportant<br>3. No opinion

	4. Important<br>5. Very Important<br>6. Not applicable
v. Improved QOL or benefit from transplant	1. Very Unimportant<br>2. Unimportant<br>3. No opinion<br>4. Important<br>5. Very Important<br>6. Not applicable
vi. Number of comorbidities	1. Very Unimportant<br>2. Unimportant<br>3. No opinion<br>4. Important<br>5. Very Important<br>6. Not applicable
vii. Cancer/Infectious history	1. Very Unimportant<br>2. Unimportant<br>3. No opinion<br>4. Important<br>5. Very Important<br>6. Not applicable
viii. Patient decision	1. Very Unimportant<br>2. Unimportant<br>3. No opinion<br>4. Important<br>5. Very Important<br>6. Not applicable
ix. Lack of access to other options (dialysis or living donor)	1. Very Unimportant<br>2. Unimportant<br>3. No opinion<br>4. Important<br>5. Very Important<br>6. Not applicable

3. **72% of your peers strongly agreed/agreed that it is appropriate to list a patient who is unlikely to outlive the graft.**

   Imagine you are evaluating a kidney transplant candidate that is unlikely to outlive the graft. Please rate the factors that would cause you to list a patient who is unlikely to outlive a graft for a transplant in terms of importance:

Factor	% of your peers who said these factors would cause them to list	Your rating on the order of importance

	**a patient who is unlikely to outlive a graft**	
Younger age	72%	1. Very Unimportant<br>2. Unimportant<br>3. No opinion<br>4. Important<br>5. Very Important<br>6. Not applicable
Health-related quality of life	76%	1. Very Unimportant<br>2. Unimportant<br>3. No opinion<br>4. Important<br>5. Very Important<br>6. Not applicable
Social Support	36%	y Unimportant<br>mportant<br>pinion<br>ortant<br>y Important<br>7. Not applicable
Symptoms on dialysis	52%	1. Very Unimportant<br>2. Unimportant<br>3. No opinion<br>4. Important<br>5. Very Important<br>8. Not applicable
Dependence in activities of daily living	28%	1. Very Unimportant<br>2. Unimportant<br>3. No opinion<br>4. Important<br>5. Very Important<br>6. Not applicable
Benefit over dialysis to patient	New factor added by participants in round 1	1. Very Unimportant<br>2. Unimportant<br>3. No opinion<br>4. Important<br>5. Very Important<br>6. Not applicable

4. **96% of your peers strongly agreed/agreed that it is appropriate to list a patient who is 65 years or older.**

   Imagine you are evaluating a kidney transplant candidate who is 65 years or older. Please rate the following factors in terms of importance:

Factor	% of your peers who said these factors would cause them to list a patient who is 65 years or older	Your Ranking
Health-related quality of life	76%	1. Very Unimportant<br>2. Unimportant<br>3. No opinion<br>4. Important<br>5. Very Important<br>6. Not applicable
Good Social Support	72%	1. Very Unimportant<br>2. Unimportant<br>3. No opinion<br>4. Important<br>5. Very Important<br>6. Not applicable
Medication adherence	64%	1. Very Unimportant<br>2. Unimportant<br>3. No opinion<br>4. Important<br>5. Very Important<br>6. Not applicable
Symptoms on dialysis	44%	1. Very Unimportant<br>2. Unimportant<br>3. No opinion<br>4. Important<br>5. Very Important<br>6. Not applicable
Dependence in activities of daily living	28%	1. Very Unimportant<br>2. Unimportant<br>3. No opinion<br>4. Important<br>5. Very Important<br>6. Not applicable
Low/No Frailty	New factor added by participant	1. Very Unimportant<br>2. Unimportant<br>3. No opinion<br>4. Important<br>5. Very Important<br>6. Not applicable

High Physical activity	New factor added by participant	1. Very Unimportant<br>2. Unimportant<br>3. No opinion<br>4. Important<br>5. Very Important<br>6. Not applicable
No medical contraindications	New factor added by participant	1. Very Unimportant<br>2. Unimportant<br>3. No opinion<br>4. Important<br>5. Very Important<br>6. Not applicable
Good functional status	New factor added by participant	1. Very Unimportant<br>2. Unimportant<br>3. No opinion<br>4. Important<br>5. Very Important<br>6. Not applicable
Low comorbidity burden	New factor added by participant	1. Very Unimportant<br>2. Unimportant<br>3. No opinion<br>4. Important<br>5. Very Important<br>6. Not applicable

5. **72% of your peers strongly agreed/agreed that it is appropriate to list a transplant candidate who is frail.**

   Imagine you are evaluating a kidney transplant candidate who is frail. Please rate the following factors in terms of importance:

**Factor**	**% of your peers who said these factors would cause them to list a patient who is frail**	**Your Rating**
Younger age	64%	1. Very Unimportant<br>2. Unimportant<br>3. No opinion<br>4. Important<br>5. Very Important<br>6. Not applicable
Health-related quality of life	64%	1. Very Unimportant

		2. Unimportant<br>3. No opinion<br>4. Important<br>5. Very Important<br>6. Not applicable
Social Support	52%	1. Very Unimportant<br>2. Unimportant<br>3. No opinion<br>4. Important<br>5. Very Important<br>6. Not applicable
Symptoms on dialysis	48%	1. Very Unimportant<br>2. Unimportant<br>3. No opinion<br>4. Important<br>5. Very Important<br>6. Not applicable
Frailty could be reversible/improved (from transplant or prehab)	New factor added by participant	1. Very Unimportant<br>2. Unimportant<br>3. No opinion<br>4. Important<br>5. Very Important<br>6. Not applicable
No medical contraindications	New factor added by participant	1. Very Unimportant<br>2. Unimportant<br>3. No opinion<br>4. Important<br>5. Very Important<br>6. Not applicable
Likelihood for success/benefit from surgery	New factor added by participant	1. Very Unimportant<br>2. Unimportant<br>3. No opinion<br>4. Important<br>5. Very Important<br>6. Not applicable

6. **68% of your peers strongly agreed/agreed that it is appropriate to list a transplant candidate who is cognitively impaired.**

   Imagine you are evaluating a kidney transplant candidate who has cognitive impairment. Please rate the following factors in terms of importance:

**Factor**	**% of your peers who said these factors would cause them to list a patient who is frail**	**Your Rating**
Social Support	72%	1. Very Unimportant<br>2. Unimportant<br>3. No opinion<br>4. Important<br>5. Very Important<br>6. Not applicable
Health-related quality of life	60%	1. Very Unimportant<br>2. Unimportant<br>3. No opinion<br>4. Important<br>5. Very Important<br>6. Not applicable
Younger age	40%	1. Very Unimportant<br>2. Unimportant<br>3. No opinion<br>4. Important<br>5. Very Important<br>6. Not applicable
Symptoms on dialysis	28%	1. Very Unimportant<br>2. Unimportant<br>3. No opinion<br>4. Important<br>5. Very Important<br>6. Not applicable
Degree of cognitive impairment (e.g. mild vs. dementia)	New factor added by participant	1. Very Unimportant<br>2. Unimportant<br>3. No opinion<br>4. Important<br>5. Very Important<br>6. Not applicable
Cognitive impairment related to ESRD/HD symptoms and not a primary neurocognitive disorder	New factor added by participant	1. Very Unimportant<br>2. Unimportant<br>3. No opinion<br>4. Important<br>5. Very Important<br>6. Not applicable
Medication adherence	New factor added by participant	1. Very Unimportant<br>2. Unimportant<br>3. No opinion

		4. Important<br>5. Very Important<br>6. Not applicable
